# Prevalence and Genetic Diversity Analysis of Human Coronavirus OC43 among Adult Patients with Acute Respiratory Infections in Beijing, 2012

**DOI:** 10.1371/journal.pone.0100781

**Published:** 2014-07-02

**Authors:** Qin Hu, Roujian Lu, Kun Peng, Xijie Duan, Yanqun Wang, Yanjie Zhao, Wen Wang, Yongliang Lou, Wenjie Tan

**Affiliations:** 1 Institute of Medical Virology, Wenzhou Medical University, Zhejiang, China; 2 Key Laboratory of Medical Virology, Ministry of Health, National Institute for Viral Disease Control and Prevention, Chinese Center for Disease Control and Prevention, Beijing, China; 3 Beijing Sixth Hospital, Beijing, China; 4 Peking Union Medical College Hospital, CAMS, Beijing, China; University of Missouri, United States of America

## Abstract

To determine the prevalence, epidemiology and genetic diversity of human coronavirus OC43 (HCoV-OC43) among adult patients with acute respiratory infections (ARI) in Beijing,five hundred and fifty-nine nasopharyngeal swab samples were collected from adult patients with ARI in Beijing. The prevalence of HCoV-OC43 infection among these patients was assessed using two different OneStep reverse transcription polymerase chain reaction (RT-PCR) assays. The epidemiological profiles of the patients with HCoV-OC43 infection were described. Partial S and N genes of HCoV-OC43 circulating strains were sequenced followed by phylogenetic analysis and amino acid alignment. Our results showed that the prevalence of HCoV-OC43 infection was 12.52% (95% CI: 9.78–15.26%), and the epidemic peak occurred in autumn. Fifty partial S and 40 partial N fragments were obtained from these patients. Phylogenetic analysis based on neighbour-joining method showed that at least three distinct clusters (A, B, C/D) of HCoV-OC43 strains were circulating among adult patients with ARI in Beijing. In addition, some novel unique clusters (UNT) of HCoV-OC43 were found in the S- and N-based phylogenetic trees. Furthermore, consensus amino acids substitutes for each cluster were also found after alignment of partial S or N sequence coding region in this study. In conclusion, we herein describe the prevalence of HCoV-OC43 among adult patients and provide substantial evidence for the genetic diversity of HCoV-OC43 circulating in Beijing.

## Introduction

Coronaviruses are ubiquitous in the environment, and they infect various animal species causing lesions of varying severity in different organs [1], [2]. The virions of coronaviruses are enveloped, roughly spherical particles that possess a linear, nonsegmented, positive-sense RNA genome of up to 32 kb [2], [3], which is the largest genome among all known RNA viruses.

To date, six species of human coronavirus (HCoVs) have been described [3]. With the exception of SARS coronavirus (SARS-CoV) (a *Betacoronavirus*) and the recent Middle East respiratory syndrome coronavirus (MERS-CoV) (a *Betacoronavirus*) [4], [5], [6], which cause severe acute respiratory syndrome, the other four common and epidemic HCoVs are HCoV-229E (an *Alphacoronavirus*), HCoV-OC43 (a *Betacoronavirus*), HCoV-NL63 (an *Alphacoronavirus*), and HCoV-HKU1 (a *Betacoronavirus*) [7]–[10]. HCoV-OC43 was first found in a patient with a cold in 1967 [8]. Virologists paid little attention to the virus, however, until SARS-CoV emerged in 2002–2003 in China [4]. Outbreaks of HCoV-OC43 occur frequently worldwide and are often associated with acute upper respiratory tract infections (URTI) [11]–[16]. Such infections might be dangerous to infants, elderly individuals, and immunocompromised patients [17], [18]. HCoV-OC43 is also suspected of causing gastrointestinal and central nervous system diseases [19]–[21].

Phylogenetic methods have enabled the classification of HCoV-OC43 as part of the 2b subgroup of *Betacoronaviruses* [4]. The time of divergence was first extensively used for coronaviruses after the SARS epidemic for estimating the date of interspecies jumping of BCoV or HCoV-OC43 ancestor from bovine to humans [22], [23]. HCoV-OC43 has a high frequency of mutation, using both base substitution and homologous RNA recombination [26], [27]. Among the structural viral proteins, the spike (S) protein is used for receptor binding and viral entry and has the most variable sequence within the coronavirus genome [24]–[27]. S protein is cleaved into two subunits, S1 and S2 [2]. The S1 gene has been reported to show the dominant genetic diversity or heterogeneity among circulating HCoV strains [24], [27]. The nucleocapsid phosphoprotein (N) gene is another common target for serological assays [28], [29]. In view of the published data and the role of structural proteins, analysis of the S and N genes would be appropriate for determination of the genetic diversity of HCoV variants. In addition, the use of multiple primer sets targeting the S and N genes is recommended for diagnosis of all types of HCoV infection [30]–[36].

The prevalence of HCoV-OC43 among respiratory tract samples from patients with ARI has been reported in China using reverse transcription-polymerase chain reaction (RT-PCR) or real-time RT-PCR assays targeting a single viral gene sequence [34], [35], [36]. Molecular epidemiology and evolution studies of HCoV-OC43 have recently been conducted in some areas [22]–[25], and the data revealed at least three distinct clusters based on RdRp, S, and N genes and the emergence of a novel genotype, D, arising from natural recombination events between genotypes B and C [25]. However, the molecular epidemiology and genetic diversity of HCoV-OC43 variants among adult patients with ARI in Beijing are not understood fully. To address these issues, this report includes investigation of the prevalence of HCoV-OC43 among 559 respiratory samples collected in Beijing as assessed using two RT-PCR assays targeting the S and N genes, respectively, followed by direct sequencing of PCR products and genetic diversity analysis of partial S and N genes of HCoV-OC43 variants. The epidemiological and clinical characteristics of patients were also evaluated and their relationships with genetic diversity determined. This is the first report of the molecular prevalence and genetic diversity of HCoV-OC43 variants among adult patients with ARI in Beijing.

## Materials and Methods

### Clinical specimens

From December 2011 to December 2012, 559 nasopharyngeal swab samples were collected from adult patients with acute respiratory tract infections (ARI) who visited the fever clinics of Peking Union Medical College Hospital and Peking Sixth Hospital. All patients over 14 years of age were selected to enroll according to a set of criteria that included respiratory symptoms, body temperature above 37.5°C, and a normal or low leukocyte count (≤10^10^/L). Clinical information and laboratory results were recorded for each patient using a standardised form. Clinical samples were placed in viral transport medium and then stored at −80°C.

All aspects of the study were performed in accordance with national ethics regulations and were approved (2010IR0128) by the Institutional Review Boards of the Centre for Disease Control and Prevention of China, as well as the Ethics Committee of Wenzhou Medical University, Peking Union Medical College Hospital or Beijing Sixth Hospital as applicable. Participants provided written informed consent regarding the purpose of the study and their right to keep information confidential. We also obtained written informed consent from the next of kin or guardians on behalf of the minors enrolled in this study.

### Nucleic acid detection and sequencing of the S and N genes of HCoV-OC43

RNA was isolated from nasopharyngeal swab specimens using a QIAamp viral RNA Mini kit (QIAGEN, Valencia, CA) according to the manufacturer’s instructions. To design primers for RT-PCR to detect HCoV-OC43, S and N gene sequences were retrieved from GenBank (http://www.ncbi.nlm.nih.gov/genbank/) and analysed using the Primer Express software version 2.0 (Applied Biosystems, Foster city, CA, USA) to allocate target sites. Screening of extracted viral RNA was conducted by OneStep RT-PCR. A 714-bp fragment (24, 204–24, 918 nt) of the S1 gene was generated using the forward primer 5′-GAACTATGGCATTTGGATACAGG-3′ and the reverse primer 5′-ATGACTGCAAATAGCCCAAATT-3′; A 749-bp fragment (29,967–30,716 nt) of the N gene was generated using the forward primer 5′-GTAAGAGAGGCCCTAATCAGAA-3′ and the reverse primer 5′- CTTCATTCATTTACTAATTACTGG-3′. The 25-µL reaction volume contained 4-µL RNA extract, 5-µL 5×QIAGEN OneStep RT-PCR Buffer, 1-µL dNTP mix (for a final concentration of 400 µM of each dNTP), 1-µL each primer (final concentration, 10 µM each), 1-µL QIAGEN OneStep RT-PCR Enzyme Mix (which contained Omniscript and Sensiscript reverse transcriptases and HotStarTaq DNA Polymerase), and RNase- and DNase-free water. The reactions were carried out with an initial 30-min reverse transcription step at 50°C, followed by a 15-min PCR activation step at 95°C, 40 cycles of amplification (30 s at 94°C, 30 s at 55°C, 1 min at 72°C), and a final 10-min extension step at 72°C. All PCR products were run on agarose gels, stained with ethidium bromide, and then visualised under UV light. Most amplicons were purified using the QIAquick Gel Extraction kit (QIAGEN) and sequenced by Cycle Sequencing Kits (Applied Biosystems) on a DNA analyser.

Specimens positive for HCoV-OC43 were tested for coinfection with other respiratory viruses, including HCoV-229E, HCoV-NL63, HCoV-HKU1, Flu A and B (influenza A and B viruses, respectively), PIV 1, 2, 3 (parainfluenza virus types 1, 2 and 3, respectively), RSV (respiratory syncytial virus), hMPV (human metapneumovirus), adenovirus and picornaviruses (including enterovirus and rhinovirus), as described previously [30], [35].

### Phylogenetic analysis and amino acid alignment

Fifty partial S gene and forty partial N gene sequences from positive samples were sequenced and submitted to the GenBank sequence database under the accession numbers KF512574 to KF512663 (**Table S1 in File S1**).

To determine the diversity of HCoV-OC43, two multiple nucleotide sequence alignments were prepared using Clustal X version 2.1 and BioEdit Sequence Alignment Editor [37]. Using MEGA 5.05 (http://www.megasoftware.net/) [38], two neighbour-joining phylogenetic trees (bootstrap values: 1000, Kimura 2-parameter model) were constructed based on alignment of the S and N nucleotide sequences [39], respectively, from the HCoV-OC43 isolates in Beijing and some HCoV-OC43 reference strains in GenBank (**Table S1 in File S1**).

Amino acid alignments of partial S and N protein sequences were performed using the BioEdit Sequence Alignment Editor and GeneDoc software (http://www.nrbsc.org/gfx/genedoc).

### Statistical analysis

Demographic data and clinical features were compared between groups of patients using the χ^2^- or Fisher’s exact test for categorical variables in SAS programme version 9.2. Logistic regression analysis was performed using 95% confidential intervals (CI) to identify any relationship between HCoV-OC43 infection and clinical symptoms. *P* values<0.05 were considered to indicate a significant difference.

## Results

### HCoV-OC43 prevalence and coinfection

Patients over 14 years old (range, 14–89 years; average, 33.43 years) were enrolled in this study (**Table 1**), comprising 47.76% (267/559) males and 52.23% (292/559) females. Among the 559 nasopharyngeal swab specimens from adult patients with ARI collected from December 2011 to December 2012, 70 (12.52%; 95% CI: 9.78–15.26%) were positive for HCoV-OC43 by either the S-target or N-target RT-PCR assay, which was significantly higher than the number determined by either assay alone, as 50 (8.94%) were positive by targeting only the S gene and 40 (7.16%) were positive by targeting only the N gene. However, statistical analysis showed no significant difference in the detection rates between the S- and N-targeting assays (8.94% vs. 7.16%; *p*>0.05). We also found that 20 (3.58%) samples were positive by targeting both S and N genes (**Table 1**).

**Table 1:** Frequency of HCoV-OC43 infection, as determined by two molecular assays, and coinfection.

HCoV-OC43 detection status	Positive Number (%)
**Different targeting assays** (from total 559 samples)	
Either S or N target detected	70 (12.52)
Only S target detected	50 (8.94)
Only N target detected	40 (7.16)
Both S and N targets detected	20 (3.58)
**Total Coinfection**	25 (35.71)
Coinfection with (from 70 samples)	
Rhinovirus	6 (8.57)
Flu A	4 (5.71)
Coxsackie virus	3 (4.29)
HCoV-HKU1	2 (2.86)
RSV	2 (2.86)
hMPV	2 (2.86)
Echovirus	2 (2.86)
Flu B	1 (1.43)
PIV 2	1 (1.43)
Poliovirus	1 (1.43)
Rhinovirus+Flu A	1 (1.43)

Coinfection with HCoV-OC43 and another respiratory virus (HCoV-HKU1, influenza [Flu] A and B viruses, parainfluenza virus [PIV] type 2, respiratory syncytial virus [RSV], human metapneumovirus [hMPV], rhinovirus, Coxsackie virus, echovirus, or poliovirus) was found in 25 patients (25/70; 35.71%; 95% CI: 24.49–46.93%), as shown in **Table 1**. The predominant viruses found in coinfections with HCoV-OC43 were rhinovirus and Flu A.

Based on analysis of clinical records, the major clinical signs/symptoms associated with presentation with HCoV-OC43 infection included fever (average temperature, 38.36°C; 97.14%; 95% CI: 93.24–101.04%), sore throat (77.14%; 95% CI: 68.96–88.18%), headache (75.71%; 95% CI: 67.30–86.98%), cough (60%; 95% CI: 48.52–71.48%), nasal stuffiness (47.14%; 95% CI: 35.45–58.83%), nasal discharge (42.86%; 95% CI: 31.27–54.45%), chills (31.43%; 95% CI: 20.55–42.31%), sputum production (18.57%; 95% CI: 9.46–27.68%), and gastrointestinal symptoms (11.43%; 95% CI: 3.98–18.88%), such as diarrhoea and vomiting (**Table S2 in File S1**). Logistic regression analysis showed that HCoV-OC43 infection was not specifically associated with gastrointestinal symptoms. Only nasal stuffiness differed significantly between the HCoV-OC43 positive and negative groups (*p* = 0.005).

### Seasonal and age-group distribution of HCoV-OC43 infection

During the study period (December 2011 to December 2012), autumn (August through October 2012) was the peak season of HCoV-OC43 infection (**Figure S1 in File S2**; *p*<0.0001). Among the 70 adult patients who were infected with HCoV-OC43, the positivity rate between males (38/267) and females (32/292) showed no significant difference. Furthermore, no significant difference in the HCoV-OC43 positivity rate was found among age groups (young, 14–40 years; middle-aged, 41–65 years; old, >65 years; **Figure S1 in File S2**).

### Phylogenetic analysis, amino acid alignment, and recombination event analysis

**Figure 1:**
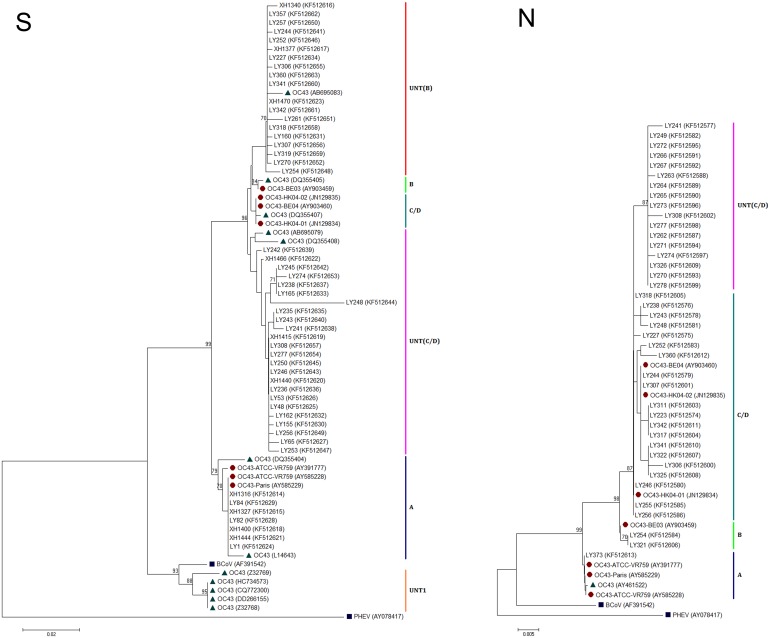
Phylogenetic analysis of partial S (left) and partial N (right) gene sequences of HCoV-OC43 isolates with respect to reference strains was performed with MEGA 5.05 using neighbour-joining. Bootstrap values were calculated from 1,000 trees. The outgroup comprised BCoV (AF391542) and PHEV (AY078417). Bootstrap values over 80% are shown. A, Cluster A; B, Cluster B; C/D, Cluster C/D; UNT, untyped Cluster (1, B and C/D); •, the sequences of reference strains cut from complete genomes found in GenBank; ▴, the sequences of reference strains cut from the partial S or N gene of HCoV-OC43 in GenBank.

The partial S and N genes of HCoV-OC43 from the nasopharyngeal swabs of patients with ARI were amplified and sequenced. The partial S gene from 50 amplicons from Beijing patients were compared with those from porcine haemagglutinating encephalomyelitis virus (PHEV), bovine coronavirus (BCoV), and 19 reference strains of HCoV-OC43 found in GenBank (**Table S1 in File S1**). The resulting phylogenetic tree revealed the existence of various virus clusters (left in **Figure 1**). Seven HCoV-OC43 strains from Beijing (LY1, LY82, LY84, XH1316, XH1327, XH1400, and XH1444) along with five reference strains from genotype A belonged to Cluster A. The OC43 (DQ355405) and OC43-BE03 (AY903459) strains of genotype B belonged to Cluster B. Genotypes C (HK04-01) and D (BE04 and HK04-02) were difficult to separate based on partial S gene sequences. Nineteen clinical isolates from Beijing patients formed a novel unique cluster and showed high similarity with isolates from Japan (AB695079) and France (DQ355408), which were named as UNT (C/D). Interestingly, two ATCC prototype strains (Z32768 and Z32769) and three English isolates (HC734573, CQ772300, and DD266155) of HCoV-OC43, which showed high similarity with BCoV (AF391542), formed a unique untyped cluster, which we termed untyped cluster I (UNT1). In addition, 20 HCoV-OC43 clinical isolates from Beijing ARI patients were phylogenetically distinct from the other major clusters and formed another unique cluster, which we termed as UNT (B).

Forty partial N-based amplicons from Beijing ARI patients combined with those from eight reference strains of HCoV-OC43 generated four major phylogenetic clusters (right in **Figure 1**). Cluster A comprised only one clinical strain from Beijing patient (LY373) and four reference strains from genotype A. Two clinical strains from Beijing (LY254,LY321) and the reference strain of genotype B (BE03) formed Cluster B. Seventeen clinical strains from Beijing (LY241, 249, 262–267, 270–274, 277, 278, 308, 326),which formed unique subcluster (UNT) and showed significant divergence from the reference strain of genotype C (HK04-01) and D (BE04, HK04-02). The remaining reference or clinical strains from Beijing formed the major cluster, named as C/D.

Multiple alignments of amino acid sequences based on partial S or N protein sequences are performed and the consensus substitutes for each cluster are summarized in **Table 2** **and** **Table 3**. Some cluster-specific markers of amino acid changes were found among partial S protein sequences of Cluster A and UNT1, with the exception of strain ATCC-VR759 (AY391777), showed a characteristic four-amino-acid deletion at the same location (amino acids 263–266). Compared to ATCC-VR759, each other cluster also showed unique amino acid substitutions at different sites, which may be considered cluster-specific markers (**Table 2**). Similar cluster-specific markers were found among the partial N protein sequences of HCoV-OC43 isolates when amino acids were aligned (**Table 3**), which showed less diversity than the analysis of S1 protein sequences.

**Table 2:** Summary on consensus amino acid changes for each cluster among S coding region of HCoV-OC43.

Cluster	Location of amino acid in S																					
	212	234	246	247	259	260	261	262	263	264	265	266	277	278	289	297	309	314	335	336	337	340
ATCC-VR759 (AT391777)	D	V	M	A	N	S	K	V	K	N	G	F	S	R	I	M	Q	P	K	L	N	N
Other A	‥	‥	‥	‥	‥	‥	‥	L	–	–	–	–	‥	‥	‥	‥	‥	‥	‥	P	‥	‥
UNT1	‥	‥	T	V	‥	‥	A	M	–	–	–	–	‥	K	V	K	L	S	I	P	‥	D
B	T	F	‥	‥	I	‥	R	R	D	I	‥	‥	‥	‥	‥	‥	‥	‥	‥	P	D	‥
C/D	T	F	‥	‥	I	‥	R	L	D	I	‥	‥	‥	‥	‥	‥	‥	‥	‥	P	D	‥
UNT(B)	T	F	‥	‥	I	‥	R	L	D	I	‥	‥	P	‥	‥	‥	‥	‥	‥	P	D	‥
UNT(C/D)	T	‥	‥	‥	I	A	R	R	N	I	‥	‥	‥	‥	‥	‥	‥	‥	‥	P	D	‥

**Table 3:** Summary on consensus amino acid changes for each cluster among N coding region of HCoV-OC43.

Cluster	Location of amino acid in N				
	374	387	401	424	441
ATCC-VR759 (AT391777)	N	M	N	R	Y
Other A	‥	‥	‥	‥	‥
B	S	‥	‥	I	‥
C/D	S	‥/V	‥	I	F
UNT(C/D)	S	‥	T	I	F

Both partial S1 and N sequence data were obtained from the same set of samples from 20 Beijing ARI patients. Our results showed that only few of 20 clinical variants belonged to the same phylogenetic clusters based on either the S or N gene (**Table S3 in File S1**). Lots of clinical strains were discrepant. These discrepancies between S- and N-based phylogenetic trees of the same clinical isolates may indicate the emergence of natural recombination events in the HCoV-OC43 population among Beijing patients with ARI. However, no demographic or clinical association with specific clusters was found (data not shown).

## Discussion

Although the human coronavirus OC43 (HCoV-OC43) was identified in 1967 [8], little information on its molecular epidemiology or phylogenetic features among adult ARI patients in Beijing is available. This study is the first report of the molecular epidemiology and genetic diversity of HCoV-OC43 infection in adult patients with ARI from Beijing. We analysed 559 nasopharyngeal swab samples, 70 (12.52%; 95% CI: 9.78–15.26%) of which tested positive for detection of HCoV-OC43, which is consistent with a previous report [34]–[36], [40]. In total, 50 amplicons from the S gene and 40 amplicons from the N gene of HCoV-OC43 isolates were sequenced. Phylogenetic tree analysis based on partial S or N gene sequences in this study showed the presence of at least three clusters (A, B, and C/D) among HCoV-OC43 strains from Beijing adult patients with ARI. Interestingly, we are the first to describe three novel HCoV-OC43 clusters or subclusters based on phylogenetic analysis of partial S genes. The UNTI cluster comprised of 4 isolates from Germany and England showed high similarity with BCoV (AF391542); however, no UNTI strain was found in our patients from Beijing. Some cluster-specific markers of amino acid changes were noted among partial S and N protein sequences from the HCoV-OC43 isolates.

Though viral isolation is the “gold standard” for laboratory diagnosis of virus infection, the great difficulty of HCoV isolation and the time-consuming nature of its culture led numerous researchers to prefer molecular detection methods, such as RT-PCR, to screen for HCoV-OC43 infection [13]–[16], [30]–[36], [40]. The analytic sensitivity of primers specific for different targets has been described previously [33], [35]. In the current study, we designed two sets of primers to amplify partial S and N gene sequences of HCoV-OC43 based on the bioinformatics literature. The detection limit for each assay was 10 copies/reaction and rate was not significantly different for the two RT-PCR assays (8.94 vs. 7.16%; *p*>0.05), but the HCoV-OC43 detection rate was enhanced significantly when the results from the two assays were combined (12.52%; 95% CI: 9.78–15.26%). This indicates that combining RT-PCR assays with different targets is advantageous for detection of HCoV-OC43 infection with increased sensitivity.

Previous reports have shown that infection with HCoV-OC43 was more common amongst elderly persons [18], [34], [40]. However, the HCoV-OC43 detection rates were similar among the different age groups of adults enrolled in our study. A few reports have suggested that the peak of infection occurs in winter and early spring [39], [40], [41]. Our study determined that HCoV-OC43 infection among adult patients occurred mainly in autumn of 2010 in Beijing, which is consistent with another study in Beijing that found that HCoV-OC43 infection was most prevalent in summer and early autumn [34]. No association was found between specific clusters of HCoV-OC43 strains and clinical signs or symptoms in this study.

The most commonly used genes used for phylogenetic studies include chymotrypsin-like protease, Pol, helicase, S, and N, because these are present in all coronavirus genomes and are of considerable length [3], [22]–[25]. Two studies from Europe analysing the S1 genes of various HCoV-OC43 strains showed high genetic diversity [26], [27]. In the current study, we constructed phylogenetic trees using the S1 sequence, which encodes the N-terminus domain (spanning amino acids 188–425) of HCoV-OC43 S and contains a hypervariable region (**Figure S2 in File S2**). Our data showed that there were at least five distinct clusters of HCoV-OC43 strains based on analysis of this partial S gene. We also constructed phylogenetic trees using the partial N sequence (spanning amino acids 288–448 of N), which represents the major diagnostic antigen and more conserved transcripts of the HCoV infection. We found that some of HCoV-OC43 isolates from our patients with ARI showed discrepancies between the S- and N-based phylogenetic trees, which might indicate the emergence of natural recombination events, as suggested in previous reports [25]–[27], since one unique feature of coronavirus replication is the high frequency of RNA recombination [25], [42]–[44]. RNA recombination is an important mechanism for the natural evolution of coronaviruses. The mean evolutionary rate of HCoV-OC43 was calculated to be 6.7×10^−4^ and 3.6×10^−4^ nucleotide substitutions per site per year for the S and N genes, respectively [24], [25]. Hence, it is easy to speculate that the appearance of a novel cluster of HCoV-OC43 strains in the current study was due to natural recombination events within the circulating HCoV-OC43 population in Beijing. To better understand the recombination and evolution of HCoV-OC43, future studies should take into account the complete genome sequences of clinical strains. That being said, the data presented here provide a better understanding of the evolution of HCoV-OC43 in different geographic areas and the role of selective pressures at different regions of the genome.

In conclusion, the prevalence of HCoV-OC43 infection among 559 adult patients with ARI in Beijing was first investigated in this study using two RT-PCR assays targeting the S and N genes. To our knowledge, these data present the genetic analysis of the largest number (70 strains) of HCoV-OC43 clinical isolates from Beijing based on partial S and N coding gene sequences. Through this analysis, we identified several novel clusters of HCoV-OC43. The most important findings of this study are that we provide substantial evidence for the genetic diversity of HCoV-OC43. At least three distinct clusters (A, B, C/D) of HCoV-OC43 strains are circulating among adults with ARI in Beijing in 2010 and some novel unique clusters (UNT) of HCoV-OC43 was found. Our data also suggest that cluster-specific genetic markers and natural recombinant events in the HCoV-OC43 genome need further investigation.

## Supporting Information

File S1 **This file contains Table S1, Table S2, and Table S3.** Table S1, Reference strains of HCoV-OC43 used in this study. Table S2, Comparison of clinical signs of those infected with HCoV-OC43 versus non-HCoV-OC43. Table S3, Information on 20 HCoV-OC43 detected using both S and N as targets.
(DOC)

File S2 **This file contains Figure S1 and Figure S2.** Figure S1, Seasonal (*p*<0.0001) and age (*p* = 0.68) distribution of HCoV-OC43 infection from December 2011 to December 2012. Figure S2,SimPlot analysis of complete genome sequence data of HCoV-OC43 strains in reference to HCoV-OC43 ATCC-VR759 (AY391777) and the location of PCR targets (S or N) in this study. The highest variability is found in the S coding region of the genome sequence.
(DOCX)
