# Supplementary material for: Prevalence and Genetic Diversity Analysis of Human Coronavirus OC43 among Adult Patients with Acute Respiratory Infections in Beijing, 2012
Source: PLoS One. 2014 Jul 2;9(7):e100781. doi: 10.1371/journal.pone.0100781 (PMC4079595; doi:10.1371/journal.pone.0100781)
Supplement: File S1 — This file contains Table S1, Table S2, and Table S3. Table S1, Reference strains of HCoV-OC43 used in this study. Table S2, Comparison of clinical signs of those infected with HCoV-OC43 versus non-HCoV-OC43. Table S3, Information on 20 HCoV-OC43 detected using both S and N as targets. (DOC) [file pone.0100781.s001.doc]

**Table S1**. Reference strains of HCoV-OC43 used in this study.

| Reference strain  (GenBank accession number) | Region | Time | Genotype | Source | Note |
| --- | --- | --- | --- | --- | --- |
| ATCC-VR759 (AY391777) | Belgium | 1967 | A | ATCC | complete genome |
| ATCC-VR759 (AY585228) | Paris | 1967 | A | ATCC | complete genome |
| OC43-Paris (AY585229) | Paris | 2001 | A | ATCC | complete genome |
| OC43 (DQ355404) | France | 2003 | A | clinical isolate | partial gene |
| OC43 (L14643) | Canada | 1967 | A | ATCC | partial gene |
| OC43 (AY461522) | USA | 2003 | A | unknown | partial gene |
| BE03 (AY903459) | Belgium | 2003 | B | clinical isolate | complete genome |
| OC43 (DQ355405) | France | 2003 | B | clinical isolate | partial gene |
| HK04-01 (JN129834) | HongKong | 2004 | C | clinical isolate | complete genome |
| BE04 (AY903460) | Belgium | 2003 | D | clinical isolate | complete genome |
| HK04-02 (JN129835) | HongKong | 2004 | D | clinical isolate | complete genome |
| OC43 (AB695079) | Japan | 2011 | NT* | clinical isolate | partial gene |
| OC43 (AB695083) | Japan | 2011 | NT | clinical isolate | partial gene |
| OC43 (HC734573) | England | 2010 | NT | unknown | partial gene |
| OC43 (CQ772300) | England | 2004 | NT | unknown | partial gene |
| OC43 (DD266155) | England | 2006 | NT | unknown | partial gene |
| OC43 (Z32768) | Germany | 1996 | NT | ATCC | partial gene |
| OC43 (Z32769) | Germany | 1996 | NT | ATCC | partial gene |
| OC43 (DQ355405) | France | 2003 | NT | clinical isolate | partial gene |
| OC43 (DQ355407) | France | 2003 | NT | clinical isolate | partial gene |
| OC43 (DQ355408) | France | 2003 | NT | clinical isolate | partial gene |

*NT, no typing.

**Table S2**. Comparison of clinical signs of those infected with HCoV-OC43 versus non-HCoV-OC43.

| Parameter | HCoV-OC43 positive | HCoV-OC43 negative | *P* |
| --- | --- | --- | --- |
|  | Number (%) | Number (%) |  |
| Total | 70 | 489 |  |
| Sex (M:F) | 38:32 (52.29/45.71) | 229:260 (46.83/53.17) | 0.24^*^ |
| Fever (≥37.3°C) | 68 (97.14) | 459 (93.87) | 0.41^**^ |
| Sore throat | 54 (77.14) | 323 (66.05) | 0.06^*^ |
| Headache | 53 (75.71) | 334 (68.30) | 0.21^*^ |
| Cough | 42 (60.00) | 235 (48.06) | 0.06^*^ |
| Nasal stuffiness | 33 (47.14) | 149 (30.47) | **0.005^*^** |
| Nasal discharge | 30 (42.86) | 163 (33.33) | 0.12^*^ |
| Chills | 22 (31.43) | 149 (30.47) | 0.87^*^ |
| Sputum production | 13 (18.57) | 121 (24.74) | 0.26^*^ |
| Gastrointestinal symptoms | 8 (11.43) | 60 (12.27) | 0.84^*^ |

M:F, male: female ratio.

* Pearson’s chi-square analysis using SAS.

** Fisher’s exact test analysis using SAS.

**Table S3.** Information on 20 HCoV-OC43 detected using both S and N as targets.

| Patient | Sex | Age | Sampling  date | Typing based on | |
| --- | --- | --- | --- | --- | --- |
|  |  |  |  | S gene | N gene |
| LY270 | F | 19 | 12 Aug.2011 | UNT(B) | UNT(C/D) |
| LY254 | M | 50 | 5 Aug.2011 | UNT(B) | B |
| LY318 | F | 27 | 13 Sep.2011 | UNT(B) | C/D |
| LY252 | M | 53 | 4 Aug.2011 | UNT(B) | C/D |
| LY244 | F | 19 | 30 Jul.2011 | UNT(B) | C/D |
| LY306 | M | 24 | 5 Sep.2011 | UNT(B) | C/D |
| LY307 | M | 29 | 5 Sep.2011 | UNT(B) | C/D |
| LY341 | F | 15 | 3 Oct.2011 | UNT(B) | C/D |
| LY342 | M | 34 | 4 Oct.2011 | UNT(B) | C/D |
| LY360 | F | 60 | 18 Oct.2011 | UNT(B) | C/D |
| LY227 | M | 22 | 20 Jul.2011 | UNT(B) | C/D |
|  |  |  |  |  |  |
| LY238 | M | 18 | 27 Jul.2011 | UNT(C/D) | C/D |
| LY243 | M | 81 | 29 Jul.2011 | UNT(C/D) | C/D |
| LY248 | M | 58 | 2 Aug.2011 | UNT(C/D) | C/D |
| LY246 | M | 21 | 31 Jul.2011 | UNT(C/D) | C/D |
| LY256 | F | 21 | 6 Aug.2011 | UNT(C/D) | C/D |
| LY241 | M | 29 | 28 Jul.2011 | UNT(C/D) | UNT(C/D) |
| LY274 | M | 15 | 13 Aug.2011 | UNT(C/D) | UNT(C/D) |
| LY277 | M | 25 | 16 Aug.2011 | UNT(C/D) | UNT(C/D) |
| LY308 | M | 34 | 6 Sep.2011 | UNT(C/D) | UNT(C/D) |

F, female; M, male.
