# Supplementary figures and images for: Prevalence and Genetic Diversity Analysis of Human Coronavirus OC43 among Adult Patients with Acute Respiratory Infections in Beijing, 2012
Source: PLoS One. 2014 Jul 2;9(7):e100781. doi: 10.1371/journal.pone.0100781 (PMC4079595; doi:10.1371/journal.pone.0100781)

Figure S1


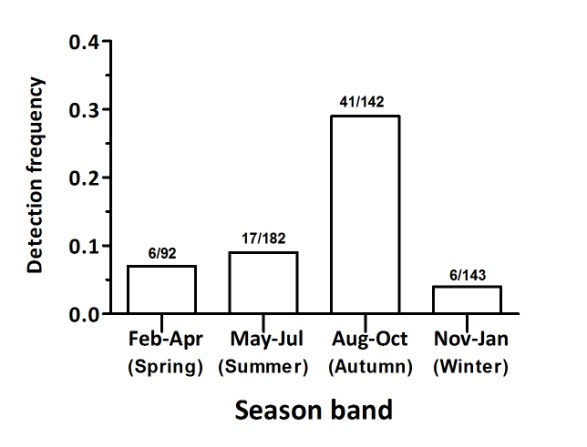

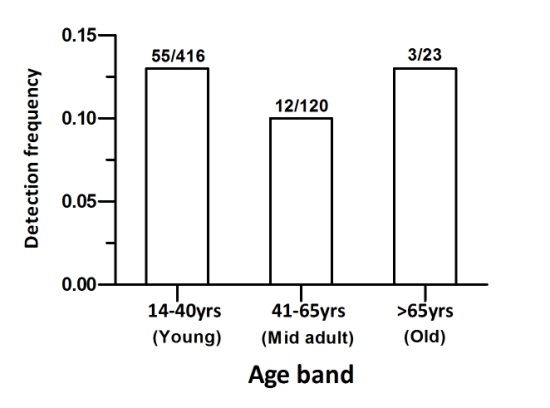


Figure S2


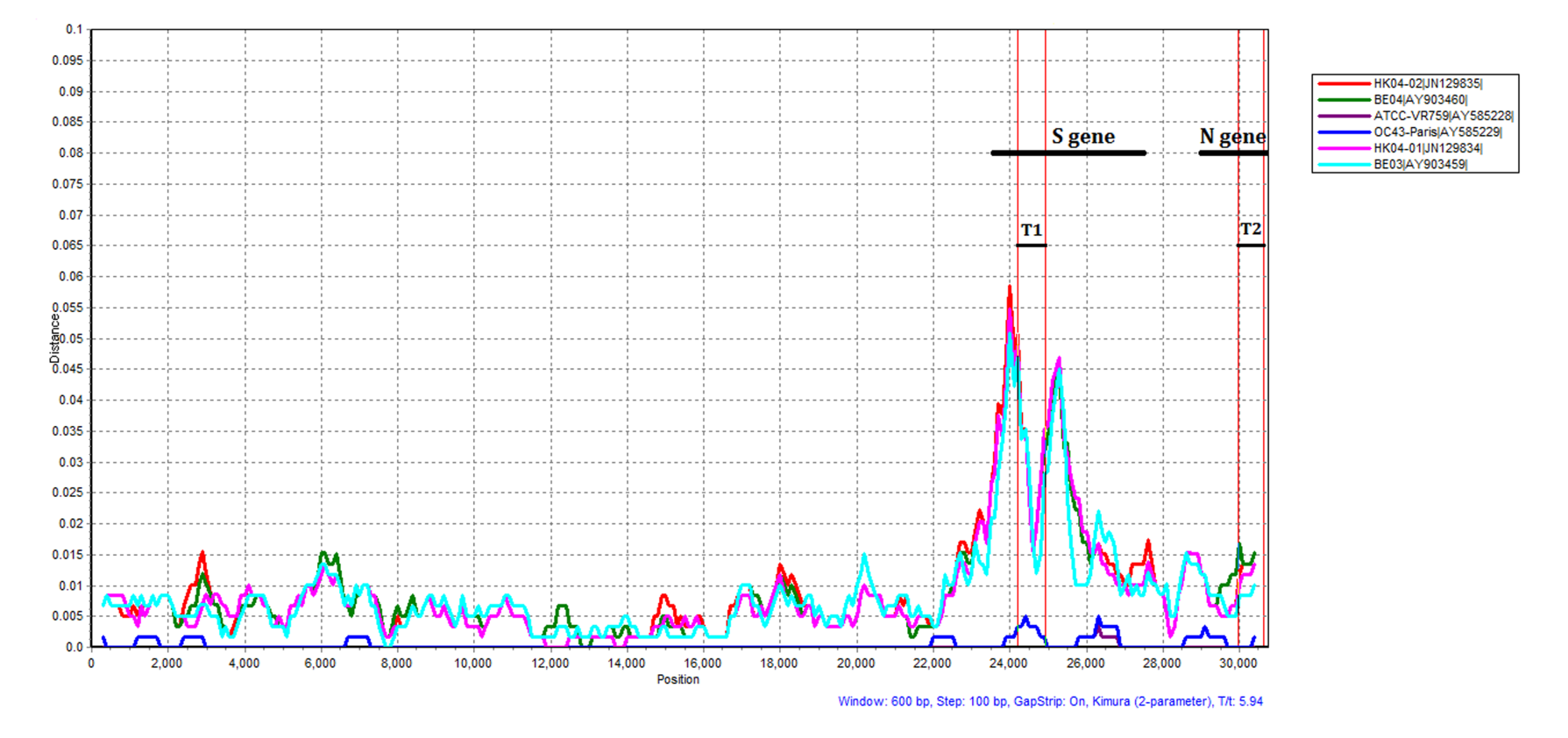

Supplement: File S2 — This file contains Figure S1 and Figure S2. Figure S1, Seasonal (p<0.0001) and age (p = 0.68) distribution of HCoV-OC43 infection from December 2011 to December 2012. Figure S2,SimPlot analysis of complete genome sequence data of HCoV-OC43 strains in reference to HCoV-OC43 ATCC-VR759 (AY391777) and the location of PCR targets (S or N) in this study. The highest variability is found in the S coding region of the genome sequence. (DOCX) [file pone.0100781.s002.docx]
